# A Review on the Weight-Loss Effects of Oxidized Tea Polyphenols

**DOI:** 10.3390/molecules23051176

**Published:** 2018-05-14

**Authors:** Dylan O’Neill Rothenberg, Caibi Zhou, Lingyun Zhang

**Affiliations:** 1 College of Horticulture Science, South China Agricultural University, Guangzhou 510640, China; Dylan.Rothenberg@colorado.edu; 2 Department of Tea Science, Qiannan Normal University for Nationalities, Duyun 558000, China; teasky@foxmail.com

**Keywords:** *Camellia sinensis*, tea catechins, obesity, weight-loss, lipid metabolism

## Abstract

The mechanistic systems in the body through which tea causes weight loss are complex and multi-dimensional. Additionally, the bioactive components in tea such as catechins, caffeine, and products of tea polyphenol oxidation vary greatly from one major tea type to the next. Green tea has been the primary subject of consideration for investigation into the preventative health effects of tea because it contains the highest levels of phenolic compounds and retains the highest antioxidant capabilities of any major tea type. However, recent research suggests decreasing body fat accumulation has little to do with antioxidant activity and more to do with enzyme inhibition, and gut microbiota interactions. This paper reviews several different tea polyphenol-induced weight-loss mechanisms, and purposes a way in which these mechanisms may be interrelated. Our original ‘short-chain fatty acid (SCFA) hypothesis’ suggests that the weight-loss efficacy of a given tea is determined by a combination of carbohydrate digestive enzyme inhibition and subsequent reactions of undigested carbohydrates with gut microbiota. These reactions among residual carbohydrates, tea polyphenols, and gut microbiota within the colon produce short-chain fatty acids, which enhance lipid metabolism through AMP-activated protein kinase (AMPK) activation. Some evidence suggests the mechanisms involved in SCFA generation may be triggered more strongly by teas that have undergone fermentation (black, oolong, and dark) than by non-fermented (green) teas. We discussed the mechanistic differences among fermented and non-fermented teas in terms of enzyme inhibition, interactions with gut microbiota, SCFA generation, and lipid metabolism. The inconsistent results and possible causes behind them are also discussed.

## 1. Introduction

Tea is a common beverage consumed daily in many parts of the world. It is classified into unfermented tea (green tea, white tea), semi-fermented tea (oolong tea) and fully fermented tea (black tea and pu’erh tea). The predominant chemical components in unfermented tea are catechins and caffeine, while in semi-fermented and fully fermented tea theaflavins, thearubigins and caffeine predominate. Catechins, caffeine and theaflavins have been confirmed to possess a broad range of biological activities.

Among the activities attributed to these bioactive compounds is the reduction of weight-gain and obesity. ‘Weight-loss teas’ currently represent a large portion of all tea sales around the world. However, the science behind tea consumption and obesity prevention is complex and constantly evolving. Some studies provide positive results, while others do not. Until recently, green tea has been the main subject of this research because it was believed that semi-fermented and fully fermented tea polyphenols are not sufficiently bioavailable to significantly affect weight gain. Recent studies have suggested that this supposition may be untrue.

In this review, we examine the possible role of tea consumption in modulating or preventing weight-gain, as well as the possible mechanisms behind the observed associations and inconsistencies. Furthermore, this review examines how fermented teas have potential to be equally or more effective compared to unfermented tea in obesity prevention. The basis for this supposition stems from approximately fifteen recently published in vivo and in vitro reviews and original research articles collected from SCI (Web of Science), Elsevier, Wiley Online Library, and Springer-Nature which collectively suggest mechanisms by which semi-fermented and fully fermented tea polyphenols might activate AMPK through SCFA generation.

Yang’s “AMPK hypothesis” proposes that AMPK activation is the main mechanism for epigallocatechin gallate (EGCG) and other catechins to influence energy metabolism [1]. The common conclusion by researchers is that the higher bioavailability of green tea catechins causes them to be more effective in AMPK activation and other systemic functions, and thus provide better health benefits. While green tea may be more effective in prevention of some diseases, new evidence suggests that obesity might be better controlled by slightly or fully fermented teas. Although we still lack in experimental evidence, recent in vivo studies have shown oolong and black tea polyphenols to be effective activators of AMPK, as well as effective enzyme inhibitors in the gastrointestinal tract. Several aforementioned in vivo studies have compared weight-loss effects of fermented teas directly in comparison to green tea, using identical extraction methods, test subjects, controlled variables, etc. We will look at the results of these studies and introduce how the data offers validity to the ‘SCFA hypothesis’. We will also give our thoughts on possible causes behind some observed inconsistencies in data and offer suggestions for future research.

## 2. Epidemiologic Evidences 

Obesity is a major health concern afflicting a wide range of countries around the world. This disease is entirely preventable with proper diet and exercise habits. Recent research has looked into the viability of plant-based phytochemicals in slowing weight gain. Results on the potential benefits of tea polyphenols on obesity and body weight reduction are summarized below (Table 1).

### 2.1. Studies in Animal Models

The anti-obesity effect of tea extract and individual tea polyphenols has been extensively studied in animal models. Most studies measure obesity-related parameters over periods of 12 weeks in mice divided into groups of high-fat diet, normal diet, and high-fat with tea added. Additionally, many animal studies include multiple tea types in order to assess the relative effectiveness of various tea types in reducing weight gain. One recent study showed that decaffeinated green and black tea polyphenols decrease weight gain in diet-induced obese mice by increasing hepatic AMPK phosphorylation and altering gut microbiota [2]. In this study, subcutaneous body fat percentage of both black and green tea groups were significantly lower than the high-fat diet group and even slightly lower than the low-fat diet group. Another study used the diet-induced obese mice model to measure at the anti-obesity effects of green compared to a Japanese dark tea known as goishi tea (like a Japanese ripe Pu’er tea) [3]. Results showed that both teas were effective in decreasing weight gain, but through different mechanisms. Green tea was more effective in slowing the fat accumulation rate, while goishi tea more effectively increased the lipolysis rate. Similar suppression of fat accumulation was seen in an in vivo experiment on rainbow trout that had 60 days green tea-supplemented diet [4].

One recent study compared the relative fat accumulation-suppressing effects of EGCG, theaflavin, and epitheflagallin (ETG, an oolong tea polyphenol) in *Drosophila melanogaster*, a multicellular organism [5]. Results showed that each tea polyphenol was effective in suppressing fat accumulation through different mechanisms, including activating different lipid-metabolising genes. While TF and ETG activated fatty acid oxidation in mitochondria, EGCG activated fatty acid oxidation in peroxisomes. There are a great number of other animal studies showing similar results of fat accumulation-suppressing properties of tea polyphenols [4,6,7,8,9,10]. The effects and mechanisms of these studies have been extensively reviewed [1,11].

### 2.2. Studies in Humans

Systematic reviews and meta-analysis covering more than 32 earlier short-term randomized trials (RCTs) indicated the beneficial effects of tea consumption in reducing body weight [12,13,14]. Among existing studies, those involving green tea or green tea catechins greatly outnumber those using fermented teas. These studies were normally 8–12 weeks, on normal weight or overweight subjects. Results showed that a catechin-caffeine mixture has a small positive effect on weight loss and weight maintenance. Moderating factors were recognized as ethnicity and caffeine tolerance. Caucasian ethnicity in addition to high regular caffeine intake both negatively moderated the weight-loss effects when compared to Asian ethnicity and low regular caffeine intake. Ethnicity is a factor due to the enzyme-related nature through which tea polyphenols induce weight loss (ethnicities vary in certain enzyme activity) [14,15].

Meta-analysis suggests that catechins and caffeine synergistically produced weight-loss effects, as opposed to the result of caffeine alone. In healthy men supplemented with green tea extract containing 270 mg EGCG and 150 mg caffeine, energy expenditure increased significantly by 4% compared with caffeine alone, and fat oxidation was 41% for green tea compared with 33% for caffeine alone (*p* < 0.01 for both) [16]. 

Some human trials have delivered negative results. Two recent studies in British adults showed no weight-loss effect from tea consumption. The first study used a relatively low dose of EGCG (200 mg daily) [17]. The second study involved daily intake of green tea (>560 mg EGCG) plus caffeine (280–450 mg) for 12 weeks [18]. In an RCT with 237 overweight women in the United States, supplementing 1315 mg of GTE (843 mg EGCG) for 12 months had no effect on body weight, BMI, or waist circumference [19]. The exact reason behind these negative results are unknown. Ethnicity-based correlations and other potential reasons for inconsistent data in human trials will be elaborated on in our discussion of inconsistent results later in this review.

## 3. Biological Mechanisms

### 3.1. Digestive Enzyme Inhibition

One of the most effective ways tea can contain obesity is in the gastrointestinal tract through the inhibition of enzymes such as pancreatic lipase, amylase, and glucosidase. By inhibiting these enzymes, tea polyphenols lower the rate of absorption of fats and sugars, thus reducing caloric intake in the body, decreasing weight-gain. 

#### 3.1.1. Pancreatic Lipase Inhibition

Pancreatic lipase is the main agent of digestion in the GI that hydrolyzes lipids (fats, oils, triacylglycerols) into monoglycerides and fatty acids, thus allowing their absorption. Inhibition of lipase in the GI is a well-known target for obesity and serves as the function of some obesity-related pharmaceuticals, such as the lipase inhibiting drug, orlistat, with inhibitory effect (IC_50_) of 0.8 µmol/L) [25]. It has been well studied that green tea polyphenols have the ability to inhibit fat digestion by way of inhibitory effect on pancreatic lipase (PL) [6,20,26,27]. The presence of a galloyl ester in green tea polyphenols is essential to efficacy as an enzyme inhibitor. It was shown that (−)-epigallocatechin-3-gallate (EGCG) inhibited pancreatic lipase in vitro (IC50 = 7.5 µmol/L) in a noncompetitive manner with respect to substrate concentration, while (−)-epigallocatechin (EGC), which has no galloyl ester, was ineffective [20,28,29]. 

Although most studies on the anti-obesity effects of tea have been conducted with green tea, there is an increasing amount of experimental evidence showing black tea polyphenols (theaflavins) might be equally or more effective [1]. One recent study showed the dose-dependent PL inhibitory effect of black tea polyphenols [25]. The galloyl ester-containing black tea polyphenols Theaflavin-3-gallate (TF3G), theaflavin-3′-gallate (TF3′G), theaflavin-3,3′-digallate (TFdiG) had in vitro inhibitory potencies of (IC50 = 4.2 µmol/L), (IC50 = 3.0 µmol/L), and (IC50 = 1.9 µmol/L) respectively. Theaflavin (TF), containing no galloyl esters, had significantly reduced PL inhibition (IC50 > 10 µmol/L). This data suggests that gallated theaflavins in Black tea have higher PL inhibition potency than EGCG in Green tea.

One oolong tea polyphenol (oolongtheanin 3′-*O*-gallate) had in vitro PL inhibition of (IC50 = 0.068 µmol/L), which is significantly more potent than EGCG, TFdiG, or even Orlistat [30]. Furthermore, these potent oolong tea PL inhibitors increased fecal lipid excretion in vivo without any side effects, unlike Orlistat [31,32,33,34]. 

Another recent study compared the effects of green tea and black tea decoctions on male Wistar rats fed high-fat diets (HFD) for 10 weeks [22]. Decoctions were made by cooking 50 g of green or black tea in 1 L of water for 15 min. This resembles a popular tea preparation method in some North African and Middle-Eastern countries. This study found that triglyceride (TG) excretion was significantly higher in both green tea decoction (GTD) and black tea decoction (BTD) than the control group (CTRL). Furthermore, TG excretion in feces was significantly higher in BTD group (230%) than in GTD group (170%) suggesting a relatively greater efficacy of BTD to reduce intestinal absorption of TG. This data suggests that although the sum of phenolic compounds in GTD decoction was greater than BTD (1236 and 435.1 mg/100 mL of decoction respectively) the relative efficiency of black tea polyphenols as enzyme inhibitors outweighed their relatively low quantity and bioavailability. 

#### 3.1.2. Glucosidase/Amylase Inhibition

Tea polyphenols not only inhibit fat-digesting enzymes, but also carbohydrate-digesting enzymes, such as amylase and glucosidase [35]. Similar to lipase inhibition, amylase and glucosidase inhibition serve to decrease energy intake, excreting carbohydrates out of the body before they can be digested. Several studies have compared glucosidase and amylase inhibitory potency among green, oolong, and black tea polyphenols. Results showed that black tea polyphenols, specifically gallated theaflavin, to be the most potent, followed by white tea, oolong tea, and green tea [36,37,38]. The results were attributed to theaflavins’ relatively high number of galloyl groups, which has been shown to be highly correlated to enzyme inhibition efficacy [36,37,39,40]. Black tea extract was shown to inhibit the degradation of disaccharides into monosaccharides by a-glucosidase in the rate small intestine. This subsequently lead to prevention of glucose absorption [41]. 

One recent study discovered C-geranylated flavanones as characteristic compounds in a Chinese (Yingde) black tea with potent glucosidase inhibition [42]. The synthesis of these unique compounds were suggested to have resulted from fermentation during black tea processing [43,44]. The newly discovered C-geranylated flavanones were tested for glucosidase inhibitory potency alongside major green tea catechins and a synthetic glucosidase inhibitor, acarbose. The most potent Yingde black tea C-geranylated flavanone had stronger glucosidase inhibitory potency (IC50 = 10.2 µmol/L) than EGCG (IC50 = 25.0 µmol/L) or the synthetic inhibitor (IC50 = 18.2 µmol/L) [42]. 

Data from the Yingde black tea study suggest that both green and black tea have compounds with potent obesity-preventing properties, however the fermentation in black tea processing can create novel compounds with even greater enzyme inhibition potency than EGCG. Additionally, the IC50 value of the a galloyl group (gallic acid) itself was found to be significantly lower (meaning more effective inhibition) than that of EC, ECG, TF, and TF1 [36]. Gallic acid is a product of catechin degradation during tea fermentation [45]. It’s strong inhibitory potency may elucidate another reason why teas that have undergone heavy fermentation (black and dark teas) are able to provide measurable weight-loss effects while containing very low amount of catechins compared to green tea.

As this paper will continue to argue, the relative potency of amylase and glucosidase inhibition among fermented teas may be particularly relevant to their weight-loss efficacy. This is because undigested carbohydrates are able to react with gut microbiota to produce short-chain fatty acids (SCFA) [46]. SCFA generation has recently been found to be capable of signaling a cascade effect in the body, activating AMPK, and inducing weight-loss.AMPK activation is important to tea-induced weight-loss, as suggested in the ‘AMPK hypothesis’ [1]. For this reason, the recent discovery of potent AMPK activation through SCFA generation in the colon may be significant in assessing how various tea types perform differently in inducing weight-loss. Relatively efficient carbohydrate inhibition shown by fermented teas leads to larger amounts of residual carbohydrates in the colon, generating higher levels of SCFA, activating more AMPK, and possibly inducing weight-loss with greater efficacy than unfermented tea. Our original ‘SCFA-hypothesis’ begins with efficient carbohydrate inhibition. 

### 3.2. Generation of Short-Chain Fatty Acids (SFCA) 

Undigested residual carbohydrates in the small intestine can function as substrate in fermentation reactions with bacteria to produce short chain fatty acids (SCFA) [47,48]. SCFA are absorbed into the blood stream where they can travel to the liver and enhance energy metabolism through mechanisms of AMPK phosphorylation and inhibition of peroxisome proliferator-activated receptor y (PPARy) [49,50]. Regulation of AMPK and PPARy can enhance fat oxidation and arrest the conversion of glucose into fat (gluconeogenesis) [51]. Addition of SCFA to the diet, resulting in energy metabolism has been shown in vivo to be beneficial in protecting against obesity and high-fat diet related diseases, like metabolic syndrome [52,53]. Furthermore, a recent in vitro study showed black tea and oolong tea polyphenols consistently outperformed green tea polyphenols in SCFA generation during anaerobic fermentation with gut microbiota over periods of 12, 24, and 36 h [24]. 

A recent in vivo study measured the effect green tea polyphenols (GTP) and black tea polyphenols (BTP) on the formation of SCFA and activation of AMPK phosphorylation [2]. Mice fed high-fat diets supplemented with GTP or BTP both saw enhanced AMPK activity, but through different mechanisms. The more bioavailable GTP were absorbed directly by organ tissue, while BTP cultivated a bacterial community in the intestines that reacted with residual carbohydrates to produce SCFA. The results showed that although GTP and BTP both significantly outperformed the control group in enhancing AMPK, BTP significantly outperformed GTP (289% and 70% AMPK increase respectively), most likely due to higher SCFA formation by BTP. This data suggests that despite lower bioavailability and lower direct absorption in organ tissue, theaflavins can still function through SCFA generation in the gut in order to meet anti-obesity targets. Authors of the study concluded that the relatively higher AMPK-enhancing effects of BTP may have been caused by stronger glucosidase and amylase inhibition, in addition to changes in the composition of the intestinal microbiota.

### 3.3. Modulation of Gut Microbiota

In recent years, an increasing amount of data has emerged examining the relationship between obesity and gut microbiota [54,55,56]. Data has shown that the composition of gut microbiota correlates highly with obesity and related diseases such as diabetes [57,58,59]. Intestinal bacteria have been shown to affect fat storage, blood-glucose balance, and hormones that affect hunger and satiety [54,60]. The two predominant phyla of gut microbial communities in humans are Firmicutes (40–60%) and Bacteroidetes (20–40%), which play critical roles in regulating fat metabolism and storage [61,62]. Studies have reported that high-fat, high-sugar diet affects gut microbiota populations by increasing the relative proportion of Firmicutes and decreasing Bacteroidetes [63,64]. Reversing the effects of high-fat diets by lowering the Firmicutes/Bacteroidetes ratio and increasing overall gut microbial diversity benefits the host and can be seen as a target in obesity prevention by tea polyphenols [65]. 

Upon consumption, most tea polyphenols (>90%) will pass through the small intestine unabsorbed, due to their low bioavailability, eventually coming into direct contact with the gut microbes, benefitting health in various ways [66,67]. The resulting interaction of gut microbiota and tea polyphenols is a complex and multidirectional metabolic energy-harvesting mechanism [68]. Gut microbes break down polyphenols into smaller, more bioavailable phenolic components, and conversely, polyphenols modulate gut microbiota communities [68,69]. 

One recent study researched the in vivo effects of (−)-epigallocatechin 3-*O*-(3-*O*-methyl) gallate (EGCG3”Me) on intestinal microbiota in C57BL/6J mice [7]. EGCG3”Me is an *O*-methylated form of EGCG that exists in limited oolong teas (due to fermentation), and has been shown to exhibit strong anti-obesity properties [70,71]. Mice in the study were fed a low fat diet (LFD), or high fat diet (HFD) with/without EGCG”Me. After 8 weeks, body weight of the HFD EGCG”Me group was significantly lower than normal HFD group. Furthermore, 8 weeks EGCG”Me treatment significantly increased the relative abundance of *Bacteroidetes*, decreased *Firmicutes*, and significantly decreased the ratio of *Firmicutes* to *Bacteroidetes* ratio from 0.55 at week 0, to 0.39 at week 8. Additionally, EGCG”Me treatment increased the the amount of short chain fatty acid (SCFA) producing bacteria, which resembled prebiotic activity by forming SCFA in the gut. This observation is concomitant with other studies reporting microbial conversion of polyphenols which affect SCFA production [72]. 

A study comparing SCFA generation by GTP and BTP [22] showed mice fed high fat, high sugar (HF/HS) diet supplemented with either GTP or BTP had microbiota shifted significantly towards a lower *Firmicutes/Bacteroidetes* ratio compared to HF/HS alone. Similar to Mei et al. [7], this study saw a strong positive correlation between weight gain and proportional presence of *Firmicutes* (*p* = 0.004), and strong negative correlation between weight-gain and proportional presence of *Bacteroidetes* (*p* = 0.004). Additionally, both studies saw a correlation between tea consumption and the increase in gut microbes responsible for SCFA generation, which has been linked to obesity prevention [73,74]. A majority of research on various tea types functioning within the gastrointestinal tract showed EGCG”Me (oolong tea fermentation product) and theaflavins (black tea fermentation product) to be more effective than unfermented (green) tea catechins in increasing gut microbiota responsible for SCFA generation. It should be noted that all teas in most cases still showed strong ability to modulate intestinal microbiota and induce a microbial environment beneficial to obesity prevention.

### 3.4. Regulating Lipid Metabolism

Fat accumulation is determined by the processes of lipolysis and lipogenesis [75]. Lipolysis is breakdown of body fat stores for energy use. Conversely, lipogenesis is the conversion of excess energy into fat for later usage. The body uses sensitive energy-sensing mechanisms (such as AMPK) to know when fat should be stored and when it should be burned for energy. Whichever teas can activate these energy sensing mechanisms more efficiently are thus capable to more effectively enhancing lipolysis and inhibiting lipogenesis. To date, most studies have shown positive correlation between tea polyphenols and modulation of these processes [1]. 

#### 3.4.1. The Role of Energy Sensing Systems

A recent review proposed that the energy-sensing molecule, AMPK, is a key mechanism that moderates energy metabolism by down-regulating lipogenesis and up-regulating lipolysis [11]. AMPK has only begun to receive attention in recent years, and much about its activation remains unknown. Several reports have implicated the role of green tea, black tea, oolong tea, and puer tea in the activation of AMPK in adipose tissue and skeletal muscle [8,76,77]. EGCG in green tea has been shown in vivo and in vitro to activate AMPK [23,77,78,79,80]. As we have reviewed, black tea consumption is capable in vivo of activating AMPK at a higher rate than green tea. This may be due to an intricate relationship of energy sensing systems in the body involving PPAR, SCFA, and AMPK.

PPAR’s belong to the nuclear receptor superfamily, and act as control switches in lipid metabolism, similar to AMPK. A recent in vivo study on male C57Bl/6J mice showed that dietary SCFA induced a PPARy-dependent switch from lipid synthesis to utilization [50]. Dietary SCFA supplementation prevented and reversed high-fat diet-induced metabolic abnormalities in mice by decreasing PPARy expression and activity. This increased the expression of mitochondrial uncoupling protein 2 and raised the AMP-to-ATP ratio, thereby stimulating oxidative metabolism in liver and adipose tissue via AMPK. These results demonstrate that SCFA act as highly effective PPARy inhibitors that are able to inhibit adipose mass increases. The ‘SCFA hypothesis’ argues that PPAR-induced AMPK activation, triggered by SCFA generation, is a critical systemic mechanism in all tea-induced weight-gain modulation. 

#### 3.4.2. Down-Regulation of Lipogenesis

PPARy is a main regulator of lipogenesis [81,82]. Suppression of PPARy stops precursor cells from differentiating into mature adipocytes [83]. One study revealed that *O*-methylated EGCG (EGCG”Me) and *O*-methylated ECG (ECG”Me), common in oolong tea, revealed higher 3T3-L1 adipocyte inhibition than their non-*O*-methylated counterparts [71]. It was concluded that this relatively efficient inhibition of adipocyte differentiation was related to the substitution of 3-OH in the d ring by methoxy groups. Additionally, it has been reported that the methylation of catechins can increase their bioavailability by facilitating their transport through cell membranes [84,85]. In this regard, *O*-methylation of tea polyphenols during oolong fermentation may lead them to outperform green tea polyphenols in inhibiting the adipocyte proliferation stage of lipogenesis.

Theaflavins have been reported to suppress the adipogenic differentiation of stem cells, the differentiation of preadipocytes to adipocytes, and the proliferation of preadipocytes. Theaflavins inhibited the differentiation of rabbit bone marrow stem cells into adipocytes at twice the rate of the control group in one in vivo study [86]. Studies have demonstrated that theaflavins were able to inhibit the proliferation and differentiation of 3T3-L1 preadipocytes and decreased the intracellular content of triglycerides [87]. The activity of theaflavins was much stronger in comparison to other tea polyphenols. In an animal study, black tea polyphenols decreased the mRNA level of three adipocyte-specific genes in the adipocyte tissue of high-fat diet-fed rats [88]. The in vivo results were a prevention of increased body weight, adipocyte tissue weight, and plasma triglycerides, suggesting an inhibition of lipogenesis.

#### 3.4.3. Up-Regulation of Lipolysis via AMPK

Tea polyphenols have been shown through AMPK activation to enhance the genes responsible for lipid catabolism. Targets of AMPK include enzymes such as fatty acid synthase (FAS) and acetyl CoA carboxylase (ACC) which are responsible for fatty acid synthesis. The resulting lowered levels of malonyl-CoA can activate CPT-1, facilitating long-chain fatty acyl CoA transport into mitochondria for beta-oxidation [89]. 

One in vivo study examined how three different tea polyphenols, EGCG, ETG, and TF enhanced expression of genes that control fatty acid oxidation in *Drosophila melanogaster*, a multicellular organism [5]. ETG (epitheaflagallin) had never been studied before, and is believed to be a catechin-derived polyphenol prominent in semi-fermented oolong tea. Results showed that ingestion of all three tea polyphenols enhanced lipid catabolism or suppressed lipid accumulation. Despite similar results, the mechanisms of fat accumulation/suppressing effects differed among TF, ETG, and EGCG. ETG was most effective in overall lipolysis rate compared to TF and EGCG. Results showed that TF and ETG activated fatty acid oxidation in mitochondria, whereas EGCG activated fatty acid oxidation in peroxisomes. This data suggests that oxidized and non-oxidized tea polyphenols work through different pathways to promote lipolysis. Furthermore, this is an example of a previously unknown tea polyphenol, ETG, derived from EGCG in the tea fermentation process, being an effective activator of lipolysis. 

Another study comparing green tea to post-fermented Japanese dark tea found that in vivo prevention of weight-gain was prevalent with both teas, but the methods of adipocyte reduction were different [3]. In this study, the total catechins in dark tea were 7 times less than green tea, and even less than standard oolong or black tea. Despite reduced catechin content, dark tea was a more effective inducer of lipolysis. The conclusion of this study suggested the relatively higher lipolysis rate in post-fermented tea was due to the presence of polyphenol metabolites, such as pyrogallol. Creation of new polyphenol metabolites during fermentation with strong lipolytic potency may explain how heavily fermented teas induce weight-loss effects comparable to catechin-rich green tea.

An in vivo study on oxidized tea polyphenols (OTP) found that OTP stimulates the biosynthesis of new mitochondria through significantly higher expression levels of *pgc1(alpha)* and *pgc1(beta)* [23]. Since mitochondria are the main site of fatty acid oxidation, the biosynthesis of mitochondria can lead to enhanced activation of fatty acid beta-oxidation. While some studies show AMPK activation as the main pathway to fatty acid oxidation, this study showed suppression of *PPARa* as the main pathway. Both pathways allow oxidized tea polyphenols to enhance fatty acid beta-oxidation in comparable or higher levels compared to green tea catechins.

## 4. Analysis of Inconsistent Results

We propose that inter-individual variation among several different systemic functions in the body may be the reason behind some of the observed inconsistencies in data. Previously we mentioned the varying degree of enzyme activity among different ethnicities. Researchers have stated that there is wide variability in flavonoid O-methylation, a major pathway of flavonoid metabolism, by the enzyme COMT [90]. The inter-individual variability of the activity of COMT could vary as much as three-fold. Furthermore, COMT enzyme activity differs between ethnic groups. Lower degrees of COMT inhibition will cause levels of norepinephrine and cAMP to rise less. Consequently, parasymphatic activity will not increase, possibly leading to less effective weight-loss results [16].

Meta-analysis revealed the importance of caffeine in the weight-loss efficacy of green tea [13]. On pooling the 6 trials in the analysis of green tea catechins (GTC) with caffeine compared with caffeine-free control, GTC ingestion significantly reduced body weight, with no effect on BMI, waist circumference, or WHR. Of the two caffeine-free trials, pooling the two trials showed no statistically significant effect. This information suggests the importance of regular caffeine intake habits with regards to weight-loss efficacy. If someone consumes high amounts of caffeine daily, then their body will be less sensitive to its stimulating effects, likely resulting in lower rates of thermogenesis and fat oxidation for that individual. 

Another relevant mechanism with high inter-individual variation is the gut microbiota [68]. For instance, one study on 58 men and women showed no effect of green tea supplementation on gut microbiota over a 12 weeks period [91]. This inconsistency may be explained by both the large variation in gut microbiota composition among individuals, and the variability of polyphenol bioavailability in the body. Microbes in the human body outnumber cells 10:1, and the polyphenol-microbiota relationship remains a relatively new topic of research. The degree of importance placed on gut microbiota with regards to weight-loss means that high variability in microflora might equate to high variability in weight-loss effects. High inter-individual variability within this complex system may explain some of the observed inconsistencies in the data. 

Dietary habits may also be partly responsible for inconsistent data. The SCFA hypothesis stresses the importance of undigested carbohydrates reacting with tea polyhphenols and gut microbiota in the large intestine to produce short-chain fatty acids (SCFA). However, a diet low in carbohydrates may lead to less substrate for SCFA generation, which could lead to lower AMPK activation. It has been shown in vitro that the production of SCFA is dramatically influenced by diet and intestinal microbiota [92]. Other recent reviews have stated how the amount and type of fiber consumed has dramatic effects on the composition of the intestinal microbiota and consequently on the type and amount of SCFA produced [93,94]. This link between diet and SCFA generation suggests that dietary habits of the individual would be a considerable factor in tea-induced weight loss. 

## 5. Conclusions

EGCG in green tea, EGCG”Me in oolong tea, theaflavins in black tea, and polyphenol metabolites in dark tea all exhibit measurable weight-loss properties in a large majority of studies. It has been long believed that green tea polyphenols are the most effective weight-loss inducers due to their higher bioavailability and strong antioxidant properties. However, recent studies are showing increasing instances of fermented tea polyphenols being equally or more effective compared to green tea polyphenols. This paper purposes a ‘SCFA hypothesis’ to explain how various tea types can all effectively induce weight-loss. The purposed pathway is the generation of SCFA in the gut resulting from a reaction between residual undigested carbohydrates, modulated gut microbiota, and tea polyphenols. According to this hypothesis, the carbohydrate inhibitory potency of the tea polyphenol, in addition to its microbiota modulations, both represent critical factors determining the ultimate weight-loss capabilities of the tea. 

We recommend more research into the area of SCFA generation by tea polyphenols/gut microbe reactions. Which specific gut microbe/polyphenol reactions maximize SCFA generation? How do dietary factors and other variables affect SCFA generation? What might be significant biomarkers of SCFA generation and resulting increased fatty acid beta-oxidation? Perhaps the profile of acylcarnitines in tea consumers change along with increased SCFA generation, and can serve as notable biomarkers of the mechanistic process. More studies in general should be done to research the reactions of tea polyphenols and gut microbiota, along with other biotransformations that coincide with the process.

Additionally, we recommend a study that measures the synergistic effects of consuming multiple tea types compared to effects of consuming one tea type. Could daily consumption of one cup each of green, oolong, and black tea outperform the effects of three cups daily of any single tea type? Might a green tea catechin, like EGCG, work synergistically with black tea theaflavins in order to produce a weight-loss effect more significant than a single tea type alone? If so, this would provide a novel insight into the weight-gain moderating properties of tea consumption. 

## Figures and Tables

**Table 1:** Epidemiologic Evidence for the association between oxidized tea polyphenols and the effect of tea on weight loss.

Test Subjects	Tea Polyphenol or Tea Type	Doses	Duration and Design	Type of Effect	Mechanisms	References
C57BL/6J mice	green tea polyphenols and black tea polyphenols (GTP)/(BTP), both decaffeinated	Average polyphenol consumption was 240 and 320 mg per kg body weight for mice fed GTP and BTP respectively	4 weeks testing period on 4 groups of mice: high-fat/high-sucrose (HF/HS), HF/HS + GTP or BTP, and low fat/high-sucrose.	GTP and BTP significantly induced weight loss. GTP and BTP induced significant increase in AMPK phosphorylation by 70 and 289% respectively.	BTPs increased pAMPK through increased SCFA production, GTPs increased AMPK in liver tissue.	[2]
3T3L-1 cells and C57BL/6J mice	Goishi tea (post-fermented tea) extract	1 mg/mL extract for cells and 4 g of tea leaves to 1 L infuse for mice	84 days testing period on 4 groups of mice, HFD(high-fat diet) tap water, HFD Goishi tea, HFD green tea.	Goishi tea is likely effective against diet-induced obesity.	Goishi tea largely influenced the reduction of serum total cholesterol and low-density lipoprotein cholesterol and inhibited oxidation.	[3]
transgenic Drosophila melanogaster	Theaflavin (TF), epitheaflagallin (ETG), and epigallocatechin gallate (EGCG)	0.1–0.5%TF, 0.1–0.5%ETG, 1–10 mM EGCG	80 days testing period on female (*n* = 140); male (*n* = 220) TF, ETG, 1 mMEGCg)	Fat accumulation-suppressing effect of ETG in Drosophila larval fat body, which was more effective than that of TF or EGCG	TF and ETG activated fatty acid oxidation in mitochondria, EGCG activated fatty acid oxidation in peroxisomes.	[5]
Male C57BL/6J mice with colonized microbial community using faecal samples from 5 volunteers	EGCG”Me (methylated EGCG found in oolong teas)	EGCG”Me was added to high fat diet at concentration of 0.1%	8 weeks study using 3 groups: (HFD), (HFD + EGCG”Me), (LFD)	Compared to HFD group, EGCG”Me group showed significantly decreased body mass gains and improved stability of gut microbiota.	EGCG”Me significantly modulated intestinal microbiota and increased production of SCFA by anaerobic microbes.	[7]
Male C57BL/6J mice	epigallocatechin gallate (EGCG)	0.2% EGCG (*w/w*)-supplemented high-fat diet for 8 weeks	a high-fat control diet and a 0.2% EGCG (*w/w*)-supplemented high-fat diet for 8 weeks	The EGCG-supplemented group showed decreased body weight gain, and plasma and liver lipids.	EGCG may have anti-obesity properties through BAT thermogenesis and mitochondria biogenesis.	[10]
10 healthy men	Green tea extract (GTE)	3 types: (50 mg caffeine + 90 mg EGCG) or (50 mg caffeine) or placebo	On 3 separate occasions, subjects were randomly assigned one of 3 treatments	GTE treatment significantly increased 24 h energy expenditure (EE) (4%: *p* < 0.01). 50 mg caffeine alone had no effects on EE.	GTE promoted fat oxidation and thermogenesis beyond that explained by it’s caffeine content alone.	[16]
High fat-fed obese C57bl/6J mice	EGCG	0.32% EGCG diet	6 weeks with mice fed high-fat diet alone or high-fat diet with EGCG	44% decrease in body weight gain in high fat-fed obese mice (*p* < 0.01).	Increased fecal lipid content by 29.4% (*p* < 0.05) compared to high-fat control.	[20]
240 men and women with visceral fat-type obesity.	Green tea with two different catechin contents	green tea containing 583 mg of catechins and 96 mg of catechins (control) per day	After a 2 weeks diet run-in period, a 12-week double-blind parallel multicenter trial was performed.	Decreases in body weight, body fat mass, waist circumference, visceral fat area, and subcutaneous fat area were greater in catechin group than control.	Further study necessary to elucidate the mechanism of action of catechins.	[21]
Male Wistar rats	15 min Green tea and Black tea decoctions brewed at 50 g tea leaves per L water (GTD)/(BTD)	GTD: 346 mg total phenolic compounds (TPC) and 73 mg caffeine BTD: 121.4 mg TPC and 89 mg caffeine	10 weeks. Three groups; high-fat diet (HFD), HFD + GTD, HFD + BTD.	Adipose tissue gains reduced by 56.4% in GTD group, 60% in BTD group. Reduction of 21 and 55% of weight gains in GTD and BTD groups.	GTD and BTD prevented fat storage in liver and lowered blood lipids by increasing fecal triglyceride excretion, with a strong effect of BTD compared to GTD.	[22]
Eight-week-old male Sprague-Dawley (SD) rats	Oxidized tea polyphenols (OTP)	Diet containing 2% OTP.	12 weeks study on three groups: LFD; HFD; HFD + OTP	OTPs significantly decreased weight gain and alleviated lipid accumulation in liver and visceral white adipose tissue. OTPs Also promoted lipid excretion.	OTP + HFD group changed expression levels of PPARs, enhanced fatty acid oxidation, and enhanced biosynthesis of mitochondria in visceral WAT.	[23]
Samples from six healthy volunteers (three females and three males, age 25–30)	Polyphenols from green tea, oolong tea, and black tea extracted with hot water (GTP, OTP, BTP)	100 g of tea powder extracted with 1600 mL of distilled water.	150 mcg of fecal mixture added to 1350 mcg of medium in anaerobic atomosphere. Samples taken at 36 h.	OTP and BTP showed better effects than GTP during fermentation. OTP performed best.	Microbes altered polyphenols to make them more bioavailable, while polyphenols proliferated SCFA-generating bacteria.	[24]
